# Supplementary material for: From sequence to enzyme mechanism using multi-label machine learning
Source: BMC Bioinformatics. 2014 May 19;15:150. doi: 10.1186/1471-2105-15-150 (PMC4229970; doi:10.1186/1471-2105-15-150)
Supplement: Additional file 2 — Java code of ml2db. Additional file ml2db_code.tar.gz contains the Java source code to run the multi-label machine learning experiments and save the results to database. The code’s Javadoc is included. [file 1471-2105-15-150-S2.zip › additional file 2/ml2db/ml2db/doc/main/ml2db/mulan/learn/crosseval/package-use.html]

Uses of Package main.ml2db.mulan.learn.crosseval


JavaScript is disabled on your browser.


- Overview
- Package
- Class
- Use
- Tree
- Deprecated
- Index
- Help

- Prev
- Next

- Frames
- No Frames

- All Classes

# Uses of Package main.ml2db.mulan.learn.crosseval

- Packages that use main.ml2db.mulan.learn.crosseval

  | Package | Description |
  | --- | --- |
  |  |  |
  | --- | --- |
  | main.ml2db.al.learning |  |
  | main.ml2db.mulan.learn.crosseval |  |
  | main.ml2db.mulan.learn.evaluation |  |
  | main.ml2db.mulan.learn.traintest |  |
  | main.ml2db.mulan.predict |  |
  | test.ml2db.mulan.learn |  |
- Classes in main.ml2db.mulan.learn.crosseval used by main.ml2db.al.learning

  Class and Description

  CrossExperimenter

  Using Mulan (multi-label machine learning), this class initialises and
  executes either Train-Test evaluation (if two different arff records are
  given) (calling `MulanTrainTestEvaluator`) or cross-evaluations (calling
  `MulanCrossEvaluator`) if only one arff record is given.

  CrossOutputManager

  Abstract class to save the output (evaluation results) of a cross or
  train-test evaluation.
- Classes in main.ml2db.mulan.learn.crosseval used by main.ml2db.mulan.learn.crosseval

  Class and Description

  CrossExperimenter

  Using Mulan (multi-label machine learning), this class initialises and
  executes either Train-Test evaluation (if two different arff records are
  given) (calling `MulanTrainTestEvaluator`) or cross-evaluations (calling
  `MulanCrossEvaluator`) if only one arff record is given.

  CrossInputManager

  An input manager for a single dataset (set of instances).

  CrossOutputManager

  Abstract class to save the output (evaluation results) of a cross or
  train-test evaluation.

  EvaluationResultsTable

  Gives the names and table field names for multi-label specific evaluation
  measures (such as micro-accuracy, macro-accuracy, hamming loss etc.)

  Folds

  Stores and delivers folds for cross validation
- Classes in main.ml2db.mulan.learn.crosseval used by main.ml2db.mulan.learn.evaluation

  Class and Description

  CrossExperimenter

  Using Mulan (multi-label machine learning), this class initialises and
  executes either Train-Test evaluation (if two different arff records are
  given) (calling `MulanTrainTestEvaluator`) or cross-evaluations (calling
  `MulanCrossEvaluator`) if only one arff record is given.
- Classes in main.ml2db.mulan.learn.crosseval used by main.ml2db.mulan.learn.traintest

  Class and Description

  CrossEvaluator

  Class created to execute cross-evaluations and extract individual evaluation
  results (not possible from the Mulan class Evaluator, because only averaged
  results are returned).

  CrossExperimenter

  Using Mulan (multi-label machine learning), this class initialises and
  executes either Train-Test evaluation (if two different arff records are
  given) (calling `MulanTrainTestEvaluator`) or cross-evaluations (calling
  `MulanCrossEvaluator`) if only one arff record is given.

  CrossInputManager

  An input manager for a single dataset (set of instances).

  CrossOutputManager

  Abstract class to save the output (evaluation results) of a cross or
  train-test evaluation.
- Classes in main.ml2db.mulan.learn.crosseval used by main.ml2db.mulan.predict

  Class and Description

  CrossExperimenter

  Using Mulan (multi-label machine learning), this class initialises and
  executes either Train-Test evaluation (if two different arff records are
  given) (calling `MulanTrainTestEvaluator`) or cross-evaluations (calling
  `MulanCrossEvaluator`) if only one arff record is given.
- Classes in main.ml2db.mulan.learn.crosseval used by test.ml2db.mulan.learn

  Class and Description

  CrossEvaluator

  Class created to execute cross-evaluations and extract individual evaluation
  results (not possible from the Mulan class Evaluator, because only averaged
  results are returned).

  CrossExperimenter

  Using Mulan (multi-label machine learning), this class initialises and
  executes either Train-Test evaluation (if two different arff records are
  given) (calling `MulanTrainTestEvaluator`) or cross-evaluations (calling
  `MulanCrossEvaluator`) if only one arff record is given.

  EvaluationResultsTable

  Gives the names and table field names for multi-label specific evaluation
  measures (such as micro-accuracy, macro-accuracy, hamming loss etc.)

- Overview
- Package
- Class
- Use
- Tree
- Deprecated
- Index
- Help

- Prev
- Next

- Frames
- No Frames

- All Classes
